# Electroacupuncture Suppresses the NF-κB Signaling Pathway by Upregulating Cylindromatosis to Alleviate Inflammatory Injury in Cerebral Ischemia/Reperfusion Rats

**DOI:** 10.3389/fnmol.2017.00363

**Published:** 2017-11-06

**Authors:** Jin Jiang, Yong Luo, Wenyi Qin, Hongmei Ma, Qiongli Li, Jian Zhan, Ying Zhang

**Affiliations:** ^1^Department of Neurology, The First Affiliated Hospital of Chongqing Medical University, Chongqing, China; ^2^Chongqing Key Laboratory of Neurology, Chongqing Medical University, Chongqing, China; ^3^Department of Integrated Chinese and Western Medicine, The First Affiliated Hospital of Chongqing Medical University, Chongqing, China; ^4^Department of Neurology, The Affiliated Hospital of Zunyi Medical College, Zunyi, China

**Keywords:** CYLD, cerebral ischemia, electroacupuncture, NF-κB signaling pathway, microglia

## Abstract

Electroacupuncture (EA) may reduce inflammatory injury by inhibiting nuclear factor-kappa B (NF-κB) signaling pathway activation after ischemic stroke. Thus, we explored temporal and spatial expression of cylindromatosis (CYLD), a negative feedback inhibitor of the NF-κB signaling pathway, to learn whether CYLD is essential for EA and reduction of inflammatory injury after focal cerebral ischemia/reperfusion. A middle cerebral artery occlusion/reperfusion (MCAO/R) model was established in male Sprague-Dawley (SD) rats and CYLD gene interference was used to investigate a potential role of neuroprotection. Rats were treated with EA (1 mA, 20 Hz for 5 min, 2 Hz for 30 min) at Baihui (GV 20), Hegu (LI 4) and Taichong (LR 3) acupoints, once daily, beginning 2 h after focal cerebral ischemia. Microglial activation and co-expression of CYLD and NF-κB were measured with immunofluorescence. Neuronal CX3CL1 expression was assayed to investigate the role of EA in the interaction between neurons and microglia via upregulation of CYLD. Then, CYLD, NF-κB p65 and p-IκBα protein expression was measured with Western blot. CYLD was mainly expressed in neurons of the peri-ischemic area after MCAO/R in rats and EA upregulated CYLD mRNA and protein from 24 to 72 h after focal cerebral ischemia/reperfusion. In addition, CYLD overexpression was positively correlated to neurobehavior and negatively connected with infarct volume and pro-inflammatory cytokines (TNF-α and IL-1β). Upregulation of CYLD by EA prevented NF-κB nuclear translocation and inhibition of neuronal CX3CL1 expression, which repressed activation of microglia. Finally, CYLD silencing significantly weakened suppression of the NF-κB signaling pathway by EA. In conclusion, upregulation of CYLD may underlie how EA could alleviate inflammatory injury after focal cerebral ischemia/reperfusion.

## Introduction

Inflammation is critical for focal cerebral ischemia/reperfusion damage and can aggravate ischemic and anoxic injury, worsening patient prognosis. The nuclear factor-kappa B (NF-κB) signaling pathway is critical for regulation of inflammation after ischemic stroke (Stephenson et al., 2000; Wang et al., 2007; Sun and Ley, 2008; Harari and Liao, 2010). The NF-κB family consists of five members, p50, p52, p65 (Rel A), c-Rel and Rel B. NF-κB prototype, which is a heterodimer composed of the Rel A (p65) and p50 subunits, is the major NF-κB protein in the nucleus. At rest, inhibitor kappa B (IκB) covers nuclear localization signal (NSL) of NF-κB that exhibits trimer (IκBα/NF-κB p65/p50 complex). Stimuli from ischemic stroke, cerebral trauma or epilepsy triggers inhibitor kappa B kinase (IKK) activation and induces phosphorylation and degradation of IκB protein, which leads to nuclear translocation of NF-κB p65/p50 and activation of NF-κB target genes (Inta et al., 2006; Yenari and Han, 2006; Yang et al., 2007). The IKK complex consists of IKKα and IKKβ and the NF-κB essential modulator (NEMO)/IKKγ (Shifera, 2010a,c; Hinz and Scheidereit, 2014). The canonical NF-κB signaling pathway associated with inflammation depends on IKKγ integrating upstream stimuli. During acute focal cerebral ischemia/reperfusion, receptor interacting protein 1 (RIP1) binds to IKKγ, contributing to IKK complex activation (Wagner, 2007; Ridder and Schwaninger, 2009; Shifera, 2010b; Courtois and Israel, 2011). Activated NF-κB induces CX3CL1 expression in neurons, which leads to activation of microglia (Sheridan and Murphy, 2013; Li et al., 2015; Liu et al., 2015), often within minutes after ischemic stroke. Activated microglia are classic (M1) and alternative (M2) activation phenotypes. M1 microglia secrete pro-inflammatory cytokines TNF-α and IL-1β, which re-activate NF-κB itself (Loddick et al., 1998; Hu et al., 2012; Benarroch, 2013; Galea and Brough, 2013; Brough and Denes, 2015; Murray et al., 2015). M2 phenotype release IL-4 and IL-10 to reduce inflammation. Previous studies show that the M1 phenotype increased for the first 24 h in the ischemic core (Morrison and Filosa, 2013; Taylor and Sansing, 2013). However, the M2 phenotype predominated in the ischemic core and the M1 type predominated in the peri-ischemic areas during the first week (Denes et al., 2007). Hence, reducing inflammatory damage of the peri-ischemic areas by suppressing the NF-κB signaling pathway during acute stage of ischemic stroke is critical.

Cylindromatosis (CYLD) negatively regulates the NF-κB signaling pathway and it is identified to be a tumor suppressor involved in familial CYLD (Hayden and Ghosh, 2008; Harhaj and Dixit, 2012; Heideker and Wertz, 2015; Ohtake et al., 2016). A recent study showed that gadolinium chloride inhibits NF-κB activation to ameliorate lung injury in rats mainly by upregulating CYLD (Zhao et al., 2017). The function of CYLD is related to deubiquitinating K63-linked chains to break RIP1 and IKKγ binding, inducing negative regulation of IKK activation (Hrdinka et al., 2016). Previous studies indicate that CYLD overexpression reduced inflammation during acute or chronic liver injury (Nikolaou et al., 2012; Hellerbrand and Massoumi, 2016). Also, CYLD negatively regulates inflammatory responses in IgA nephropathy (Cui et al., 2009). Thus, CYLD may be a potential endogenous regulator against inflammatory injury. Recently, CYLD was shown to negatively regulate necroptosis induced by oxygen-glucose-deprivation (OGD) in primary cortical neurons (Feng et al., 2017). However, CYLD studies are limited that describe the effects on inflammatory injury during focal cerebral ischemia/reperfusion in rats.

Electroacupuncture (EA), a non-drug treatment, has been often studied in China (Wang et al., 2010; Lu et al., 2013; Lin et al., 2016; Xie et al., 2016; Chi et al., 2017; Zhu X. L. et al., 2017; Zhu Y. et al., 2017). EA is often used as an adjuvant therapy for treating ischemic stroke and inflammatory diseases in China (Kim et al., 2007; Yang et al., 2010; Liu A. J. et al., 2015; Ratmansky et al., 2016). A previous study suggests that EA applied to Quchi (LI11) and Zusanli (ST36) acupoints regulated the miR-9-mediated NF-κB signaling pathway and reduced secretion of TNF-α and IL-1β after ischemic stroke (Liu et al., 2016b). We reported that EA reduced expression of IKKα and IKKβ and may restrain function of them to regulate activation of the NF-κB signaling pathway and increased anti-inflammatory cytokines (such as IL-4 and IL-10) and ameliorate inflammation during acute stages of focal cerebral ischemia/reperfusion (Lu et al., 2013; Qin et al., 2013; Yang et al., 2013). Subsequently, we found that EA regulated the NF-κB signaling pathway to ameliorate inflammatory injury in a focal cerebral ischemia/reperfusion model rats by upregulating A20 (Zhan et al., 2016). CYLD and A20 are subclasses of the deubiquitinase (DUB) family. CYLD and A20 may regulate initial and resolving stages of NF-κB activation (Lork et al., 2017). Thus, EA may inhibit NF-κB activation via regulating CYLD. However, this is associated with CYLD expression is not clear. Thus, we studied the spatiotemporal characteristics of CYLD and the effects of EA on CYLD expression and possible mechanism(s) by which EA may regulate the NF-κB signaling pathway via CYLD to reduce neuro-inflammation. Moreover, we explored the role of CYLD in regulating interactions between neurons and microglia in neuro-inflammation induced by focal cerebral ischemia/reperfusion.

## Materials and Methods

All studies were performed following an experimental protocol approved by the Ethics Committee for Animal Experimentation of Chongqing Medical University, and in accordance with the guidelines of the National Institutes for Animal Research. The experimental process followed randomized and blinded guidelines.

### Animal Model

Specific pathogen-free (SPF) male Sprague-Dawley (SD) rats (280–300 g), were purchased from the Experimental Animal Center of Chongqing Medical University. Temperatures were monitored rectally and maintained between 36.5–37.5°C with a thermostatic pad. Rats were anesthetized with 3.5% chloral hydrate (1 ml/100 g, ip). The right common and right external carotid artery were exposed using a ventral midline neck incision. An ischemic stroke model was established by inserting a 2.0 monofilament nylon suture (Ethicon Nylon Suture; Ethicon Inc., Osaka, Japan) into the right external carotid artery and extending it to the beginning of the middle cerebral artery (Longa et al., 1989). Rats were anesthetized with 3.5% chloral hydrate (0.3 ml) to relieve pain when the nylon monofilament was withdrawn. Reperfusion was accomplished by withdrawing the suture after 2 h of ischemia. Then, the incision was sutured and sterilized. The same operation was performed for sham rats, except for insertion of the monofilament nylon suture. In addition, regional cerebral blood flow (rCBF) was monitored using a disposable microtip fiber optic probe (diameter 0.5 mm) attached to the skull through a perforation and connected to a laser Doppler computerized main unit (PeriFlux 5000, Perimed AB, Sweden). A successful MCAO model was confirmed when rCBF decreased to 20% and recovered to >80% of baseline (pre-ischemia) level. Rats not meeting the standard were excluded from the experiment.

### Lentivirus Production and Administration

Lentivirus carrying exogenous CYLD (LV-CYLD, 2 × 10^8^ transduction unit, TU/ml) and CYLD shRNA (LV-shCYLD, 3 × 10^8^ transduction unit, TU/ml) for overexpressing and silencing CYLD, respectively, and control (LV-control) were purchased from Genechem (Shanghai, China) and stored at −80°C until use. Lentiviruses were immediately centrifuged and placed on ice prior to injection and the viral dose was determined according to an 80% brain infection rate. Two weeks before surgery, rats received an intracerebral ventricular injection of 5 μl LV-CYLD, LV-shCYLD, or LV-control. The rats were anesthetized as described above and placed onto a stereotaxic frame (Stoelting, Wood Dale, IL, USA). According to stereotactic parameters and rat size, a cranial hole, located 1.3 mm lateral and 1.5 mm posterior to the bregma, was drilled on the right hemisphere and the right lateral ventricle to a depth of 3.8 mm beneath the dural surface was slowly inserted. Then, lentivirus (LV-CYLD or LV-shCYLD) or vehicle (LV-control) was injected into the right lateral ventricle (0.5 μl/min). Gene interference on CYLD overexpression or silencing was verified via immunofluorescence.

### Electroacupuncture Treatment (EA)

**Figure 1:**
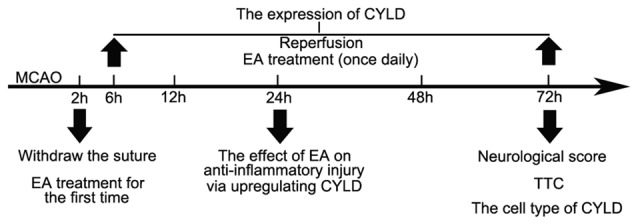
The timeline of experiment. The nylon monofilament was withdrawn after 2 h of ischemia. Rats were initially treated with electroacupuncture (EA) when the nylon monofilament was withdrawn and then once daily until sacrifice. Rats in MCAO/R + EA, MCAO/R + LV-CYLD + EA, MCAO/R + LV-shCYLD + EA, MCAO/R + vehicle + EA groups except MCAO/R group were treated with EA. The characteristics of cylindromatosis (CYLD) expression were measured after reperfusion (at 6, 12, 24, 48, 72 h). The effect of EA on inflammatory injury was investigated at 24 h reperfusion. Neurological scores and 2,3,5-triphenyltetrazolium chloride (TTC) and the cell type of CYLD were tested at 72 h reperfusion.

A Baihui (GV 20) acupoints (intersection of the sagittal midline and the line between the ears), a Hegu (LI 4) acupoint (the radial side of the left second metacarpal midpoint) and a Taichong (LR 3) acupoint (the dent between the first and second left metatarsal) were selected according to the Experimental Animals Meridians Mapping. Rats were stimulated at an intensity of 1 mA, with a frequency of 20 Hz, for 5 min followed by 2 Hz for 30 min, using a G6805-2 EA Instrument (Model no. 227033; Xinsheng Ltd., Qingdao, China; Zhan et al., 2016). Rats were initially treated with EA when the nylon monofilament was withdrawn and then once daily until sacrifice (Figure 1). Rats in MCAO/R + EA, MCAO/R + LV-CYLD + EA, MCAO/R + LV-shCYLD + EA, MCAO/R+vehicle + EA groups except MCAO/R group were treated with EA.

### Neurobehavioral Evaluation

Rats were assessed neurologically by a researcher blinded to animal treatment at 6, 12, 24, 48 and 72 h of reperfusion. An 18 point system (Supplementary Table S1) was used according to methods described by Garcia’s group (Garcia et al., 1995).

### Infarct Volume Assessment

Rats were anesthetized as described and rapidly beheaded after 72 h of reperfusion. The intact brain was immediately frozen at −20°C for 20 min and then sliced into 2-mm thick coronal sections which were stained with 2% 2,3,5-triphenyltetrazolium chloride (TTC, Sigma-Aldrich, St. Louis, MO, USA) for 15 min at 37°C (Bederson et al., 1986). Images of brain slices were collected using a digital camera and uploaded for analysis. The volume of pale brain areas were regarded as infarcts and were measured using imaging analysis software (Image J) by a researcher blinded to animal treatment groups. Infarction volume was shown as percent of intact hemispheres.

### Real-Time Quantitative qPCR

Total RNA was extracted from peri-ischemic areas of brain tissue (Supplementary Figure S1) with Trizol (TaKaRa Biotechnology Co, Japan) at different times. RNA was reverse transcribed into cDNA with a PrimeScript RT reagent kit with gDNA Eraser (TaKaRa). RT-qPCR was performed with the iQ5 Gradient Real-Time PCR detection system (Bio-Rad Co., city and state required) with SYBR Green (SYBR Premix Ex TaqII, TaKaRa) for Bcl-2 related proteins A1α (Bcl2a1a) and CYLD (Supplementary Table S2). The following cycling conditions were used: (1) 30 s at 95°C; (2) 5 s at 95°C; (3) 30 s at 60°C; (4) 30 s at 72°C, Plate read; (5) Go to (2) 39 cycles; (6) 10 s at 95°C; and (7) Melt curve 60°C to 95°C, increments of 0.5°C. Then, plates were read. The melting curve of each sample was analyzed to determine primer-target specificity. Data were normalized to the mean of β-actin. Relative mRNA expression was calculated using the 2^−ΔΔCt^ method.

### Double-Immunofluorescent Labeling

Rats were deeply anesthetized with 3.5% chloral hydrate at 24 h after reperfusion. Bodies were then perfused with saline, followed by 4% paraformaldehyde (PFA, 4°C). Intact brains were rapidly harvested, post-fixed in 4% PFA for 48 h, fully dehydrated in 30% sucrose, and cut into 10-μm thick continuous coronal brain slices. The sections were then processed by rupturing membranes with 0.3% Triton X-100 for 20 min at 37°C and blocked with 5% goat or donkey serum for 1 h at 37°C. Sections were subsequently incubated with prepared primary antibodies at 4°C overnight as follows: Anti-NeuN mouse (labeled neurons, MAB377, Millipore, 1:100), anti-GFAP mouse (labeled astrocytes, BM0055, Boster, 1:100), anti-Iba1 goat (to label microglia, NB100-1028SS, Novus, 1:50), anti-CYLD rabbit (11110-1-AP, Proteintech, 1:100), anti-CYLD mouse (SC-74435, Santa Cruz, 1:100), anti-NF-κB p65 rabbit (#8242, Cell Signaling Technology, 1:50) and anti-CX3CL1 rabbit (ab25088, Abcam, 1:100). After rewarming for 1 h at 37°C, sections were incubated with secondary antibodies for 1 h at 37°C in the dark: Alexa Fluor 594-conjugated goat anti-rabbit IgG (H + L) (SA00006-4, Proteintech, 1:200), Alexa Fluor 594-conjugated donkey anti-rabbit IgG (H + L) (SA00006-8, Proteintech, 1:200), Alexa Fluor 488-conjugated goat anti-mouse IgG (H + L) (SA00006-1, Proteintech, 1:200), FITC-conjugated Affinipure donkey anti-goat IgG (H + L) (SA00003-3, Proteintech, 1:200). Finally, nuclei were stained with DAPI (C1005, Beyotime Biotechnology) for 5 min at room temperature. All images were captured using an immunofluorescent microscope (Nikon A1R Confocal Microscope).

To quantify the immunofluorescent labeled CX3CL1 expression and nuclear NF-κB p65 expression under CYLD silencing, neuronal CX3CL1 and nuclear NF-κB p65 positive cells were counted at the border of the ischemic cortex. Counts were expressed as numbers/mm^2^ of images with 400× magnification. Five fields of the ischemic cortex were randomly chosen for every group. Imaging analysis software (Image J) was used to count positive cells by a researcher blinded to treatments.

### Western Blot

Rats were treated as described and subsequently perfused with saline. Tissues were extracted from the peri-ischemic areas of brain (Supplementary Figure S1). Tissues were homogenized in RIPA lysis buffer (no. P0013B, Beyotime) with PMSF (Beyotime, Shanghai, China) and additional phosphatase inhibitors for detecting phosphorylated IκBα (p-IκBα). After centrifugation at 12,000 rpm for 15 min, total protein was extracted from supernatants on ice and the cytoplasmic/nuclear proteins were extracted using a nuclear and cytoplasmic protein extraction kit (no. P0027, Beyotime). Western blot was performed as described previously (Bio-Rad Co., city and state required; Zhan et al., 2016). Briefly, 50–100 μg protein was separated with 8%–12% SDS-PAGE (Beyotime) and then was transferred to PVDF membranes. After blocking nonspecific epitopes, membranes were incubated with primary antibodies at 4°C overnight, as follows: anti-CYLD rabbit (11110-1-AP, Proteintech, 1:2000), anti-NF-κB p65 mouse (#6956, Cell Signaling, 1:1500), anti-IκBα rabbit (18220-1-AP, Proteintech, 1:1000), anti-phospho-IκBα rabbit (#2859, Cell Signaling, 1:1000). Membranes were then incubated with corresponding secondary antibodies, horseradish peroxidase-conjugated goat anti-rabbit (Abclonal, 1:3000) or goat anti-mouse (Abclonal, 1:3000) for 2 h at 37°C. Immune blots were scanned using a gel imaging apparatus (Vilber Lourmat fusion FX 7 Spectra, France) and analyzed with appropriate analysis software (FUSION-CAPT, France).

### Enzyme-Linked Immunosorbent Assay (ELISA)

Tissues were extracted from peri-ischemic cortices of brains. TNF-α and IL-1β in cortical homogenates were measured with Enzyme-linked immunosorbent assay (ELISA) (no. SEA133Ra 96T and SEA563Ra 96T, Cloud-Clone Corp., Katy, TX, USA) according to the manufacturer’s instructions. To each sample was added 100 μl standard and samples were incubated 1 h at 37°C. Supernatant was aspirated and to each sample was added 100 μl prepared detection reagent A. Samples were incubated 1 h at 37°C, and supernatant was aspirated and samples were washed three times. To each sample was added 100 μl prepared detection reagent B and samples were incubated 30 min at 37°C. Supernatant was aspirated again and samples were washed 5 times. To each sample was added 90 μl substrate solution and samples were incubated 10–20 min at 37°C. Then 50 μl stop solution was added to samples and plates were read at 450 nm immediately.

### Statistical Analysis

GraphPad Prism Version 6.0 was used for all statistical analyses. Neurological deficit scores are presented as medians (interquartile ranges) and analyzed using Kruskal-Wallis tests followed by *post hoc* Dunn’s multiple comparison tests. All other data are expressed as means ± SEMs. The effect of EA on CYLD mRNA or protein was compared among groups using two-way analyses of variance (ANOVA) with Bonferroni *post hoc* tests. Other data were analyzed by one-way ANOVA to analyze intergroup differences. *p* < 0.05 was considered statistically significant.

## Results

### CYLD Protein Expression after Focal Cerebral Ischemia/Reperfusion

**Figure 2:**
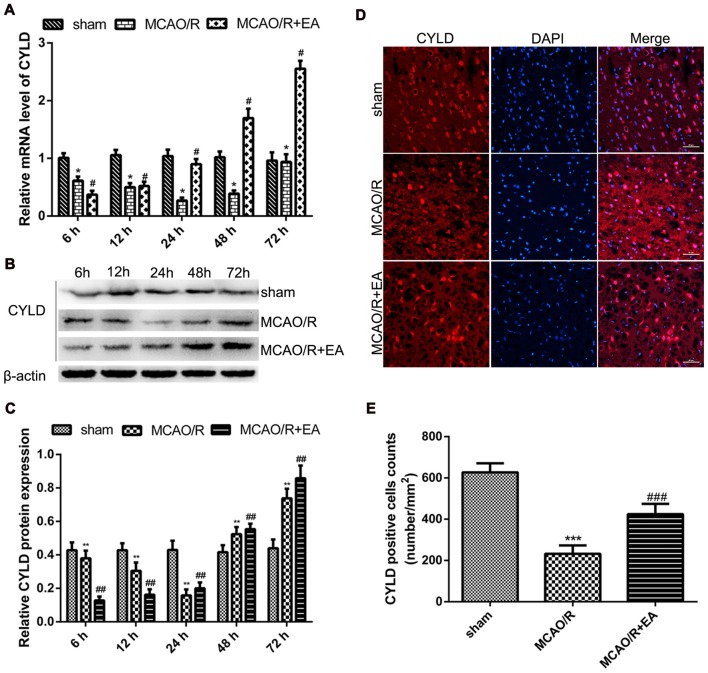
EA upregulated CYLD mRNA and protein after focal cerebral ischemia/reperfusion. **(A)** CYLD mRNA measured with RT-qPCR at 6, 12, 24, 48, 72 h reperfusion in the border region of the ischemic cortex in MCAO/R and MCAO/R + EA groups and sham cortices. Relative mRNA normalized to β-actin. Sham was the negative control. *p < 0.001 vs. sham, ^#^*p* < 0.001 vs. MCAO/R group, *n* = 5/group.** (B)** Western blot of CYLD expression at 6, 12, 24, 48, 72 h reperfusion in the ischemic cortical border. **(C)** Relative protein expression normalized to β-actin showed that CYLD expression increased with EA from 24 to 72 h after reperfusion. **p < 0.05 vs. shams, ^##^*p* < 0.05 vs. MCAO/R group, *n* = 5/group. **(D)** Immunofluorescent staining showed expression of CYLD at 24 h reperfusion at the ischemic cortical borders in MCAO/R and MCAO/R + EA groups and sham cortices, *n* = 5/group (Scale bar = 50 μm). **(E)** The CYLD-positive cell counts at 24 h reperfusion at the ischemic cortical borders of MCAO/R and MCAO/R + EA groups and corresponding area in the sham. CYLD-positive cells expressed as number/mm^2^. ***p < 0.05 vs. sham, ^###^*p* < 0.001 vs. MCAO/R group, *n* = 5/group.

To explore CYLD expression after ischemic stroke and the effect of EA on CYLD. The level of CYLD mRNA and protein were tested by RT-qPCR and western blot respectively after 6, 12, 24, 48 and 72 h reperfusion. Rats were randomly divided into three groups: sham, MCAO/R and MCAO/R + EA. Figure 2A shows that CYLD mRNA was lowest after 24 h reperfusion in the MCAO/R group (Figure 2A) and this increased at 48 h and peaked after 72 h reperfusion. CYLD mRNA increased from 12 to 72 h in the MCAO/R + EA group compared with the MCAO/R group (Figure 2A). There was a little CYLD protein expression in the sham group (Figures 2B,C). Similarly, CYLD expression was lowest after 24 h reperfusion and increased after 48 h reperfusion in the MCAO/R group. CYLD protein peaked after 72 h reperfusion in the MCAO/R group compared with the sham group (Figures 2B,C). CYLD protein significantly increased from 24 to 72 h after reperfusion in the MCAO/R + EA group compared with the MCAO/R group (Figures 2B,C).

Spatial expression of CYLD in brain tissues was measured at 24 h reperfusion with immunofluorescence. CYLD expression was found in cortex and hippocampus of sham brains (Supplementary Figure S2). After 24 h reperfusion, CYLD positive cells significantly decreased in the border region of ischemic areas in the MCAO/R group compared with sham (Figures 2D,E). CYLD-positive cells increased after EA treatment in the MCAO/R + EA group compared with the MCAO/R group (Figures 2D,E).

### CYLD Is Expressed in Cortical Neurons after Focal Cerebral Ischemia/Reperfusion

**Figure 3:**
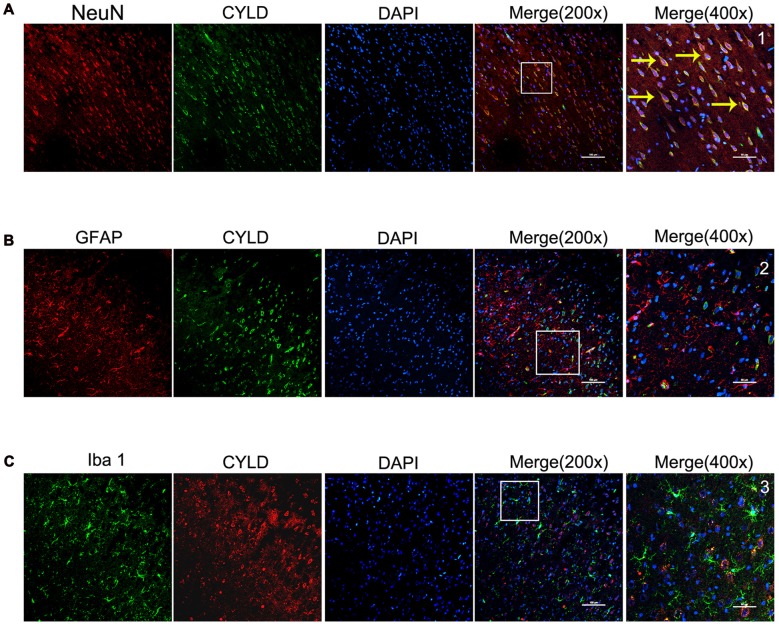
Spatial distribution and cell type of CYLD ischemic cortical borders after 2 h of focal cerebral ischemia and 72 h of reperfusion, *n* = 6 (Scale bar = 100 μm). **(A)** Co-expression of CYLD (green) and NeuN (red, neurons). **(B)** Microglia co-expressing CYLD (green) and Iba1 (red, microglia).** (C)** Astrocytes co-expressing CYLD (green) and GFAP (red, astrocytes). 1–3 were magnified from a merged image and show CYLD. Yellow arrows indicate co-expression between CYLD and neurons (Scale bar = 50 μm).

We investigated the main cell type expressing CYLD and the spatial expression in the peri-ischemia areas of rats after 72 h reperfusion using double-immunofluorescence labeling staining. CYLD protein was mainly expressed in cortical neurons of peri-ischemic areas after focal cerebral ischemia/reperfusion (Figure 3A), but there was little co-expression with Iba1 or GFAP (Figures 3B,C). Thus, CYLD immunopositive neurons may be key to reducing inflammatory injury after focal cerebral ischemia/reperfusion in rats.

### CYLD Suppresses Inflammatory Injury after Focal Cerebral Ischemia/Reperfusion

**Figure 4:**
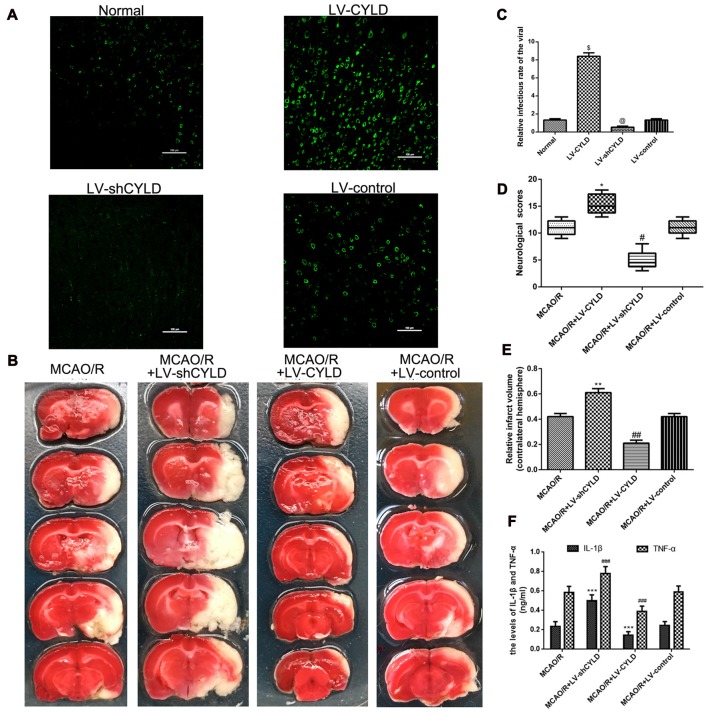
CYLD plays neuroprotective and anti-inflammatory in the peri-ischemic area after focal cerebral ischemia/reperfusion. **(A)** Immunofluorescence of gene interference of CYLD overexpression or silencing (green, CYLD) (Scale bar = 100 μm).** (B)** Relative infectious rate of viral. Normal was the negative control. ^@^*p* < 0.05, ^$^*p* < 0.001 vs. Normal, *n* = 5/group.** (C)** Neurological scores evaluated at 72 h reperfusion in MCAO/R, MCAO/R + LV-shCYLD, MCAO/R + LV-CYLD, MCAO/R + LV-control groups. *p < 0.001, ^#^*p* < 0.001 vs. MCAO/R group, *n* = 10/group.** (D)** Infarct volumes are percent of intact hemispheres at 72 h reperfusion. **p < 0.001, ^##^*p* < 0.001 vs. MCAO/R group, *n* = 10/group.** (E)** Images of cerebral infarction stained with 2% TTC (white, infarct tissue; red, non-infarct tissue) at 72 h reperfusion. **(F)** IL-1β and TNF-α in ischemic cortical border regions by enzyme-linked immunosorbent assay (ELISA) at 72 h reperfusion. ***p < 0.05, ^###^*p* < 0.05 vs. MCAO/R group, *n* = 4/group.

To investigate the effect of gene interference on CYLD overexpression or silencing, the CYLD protein expression was tested by immunofluorescence from four groups: Normal, LV-shCYLD, LV-CYLD and LV-control. CYLD positive cells were significantly decreased with LV-shCYLD compared with the normal group (Figures 4A,B). CYLD positive cells in the LV-CYLD group were significantly increased compared to the normal group (Figures 4A,B).

To further explore the effect of CYLD on inflammatory injury during ischemic stroke, we used neurological scores, TTC staining and ELISA at the indicated time. Animals were divided into four groups: MCAO/R, MCAO/R + LV-shCYLD, MCAO/R + LV-CYLD, MCAO/R + LV-control. Neurological scores are not different before 48 h reperfusion (data not shown). After 72 h reperfusion, neurological function for the MCAO/R + LV-CYLD group was improved compared with the MCAO/R group (Figure 4C). However, compared with the MCAO/R group, rat neurological function was significantly reduced in the MCAO/R + LV-shCYLD group (Figure 4C). There was no significant difference between MCAO/R + LV-control and the MCAO/R group (Figure 4C). Moreover, the infarct volume was smaller in the MCAO/R + LV-CYLD group compared to the MCAO/R group (Figures 4D,E). Rat infarct volumes in the MCAO/R + LV-shCYLD group were larger than those in the MCAO/R group 72 h after reperfusion (Figures 4D,E). There was no significant difference in infarct volume between MCAO/R + LV-control and the MCAO/R group (Figures 4D,E). Thus, CYLD overexpression may play a significant neuroprotective role after focal cerebral ischemia/reperfusion.

ELISA showed that TNF-α and IL-1β in the border region of ischemic areas of rats after MCAO/R + LV-CYLD were significantly decreased compared with MCAO/R 24 h after reperfusion (Figure 4F). TNF-α and IL-1β with MCAO/R + LV-shCYLD were increased compared with MCAO/R (Figure 4F). There was no significant difference between MCAO/R + LV-control and MCAO/R (Figure 4F). Thus, overexpression of CYLD protein had anti-inflammatory effects by reducing TNF-α and IL-1β, which damage brain tissue after focal cerebral ischemia/reperfusion. Silencing CYLD expression exacerbated stroke outcomes.

### CYLD Silencing Partially Weakens Neuroprotective Effects of EA after Focal Cerebral Ischemia/Reperfusion

**Figure 5:**
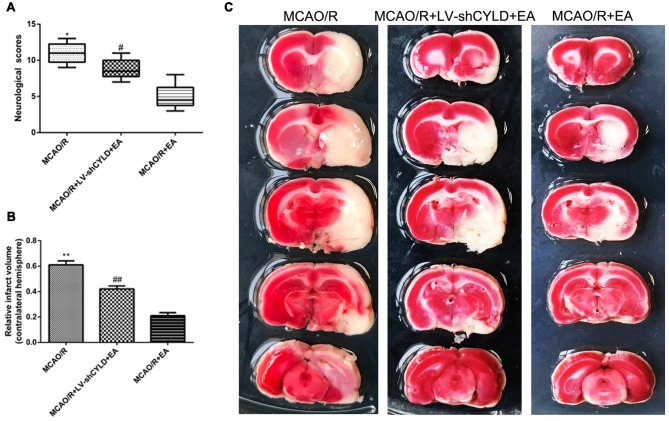
CYLD silencing partially weakens neuroprotective effects of EA after focal cerebral ischemia/reperfusion. Neurological scores and infarct volumes at 72 h in MCAO/R, MCAO/R + LV-shCYLD + EA and MCAO/R + EA groups. **(A)** Neurological scores are medians (interquartile range), *p < 0.001, ^#^*p* < 0.05 vs. MCAO/R + EA group, *n* = 10/group. **(B)** Infarct volume as percent of intact hemispheres. **p < 0.001, ^##^*p* < 0.001 vs. MCAO/R + EA group, *n* = 10/group. **(C)** Images of cerebral infarction stained with 2% TTC (white, infarct tissue; red, non-infarct tissue) at 72 h reperfusion.

To explore whether CYLD is required for the neuroprotective effect of EA, neurobehavioral evaluations and infarct volume were measured in the three groups (MCAO/R, MCAO/R + LV-shCYLD + EA and MCAO/R + EA) after 72 h reperfusion. After 72 h reperfusion, neurologic function improved (Figure 5A) and smaller infarct volume (Figures 5B,C) was noted with MCAO/R + EA compared with MCAO/R. In contrast, poor neurologic function (Figure 5A) and a larger infarct volume (Figures 5B,C) was seen with MCAO/R + LV-shCYLD + EA compared to MCAO/R + EA. Thus, CYLD silencing partially weakens any neuroprotective effects of EA.

### CYLD Silencing Weakens the Effect of EA on Anti-Inflammatory Injury after Focal Cerebral Ischemia/Reperfusion

**Figure 6:**
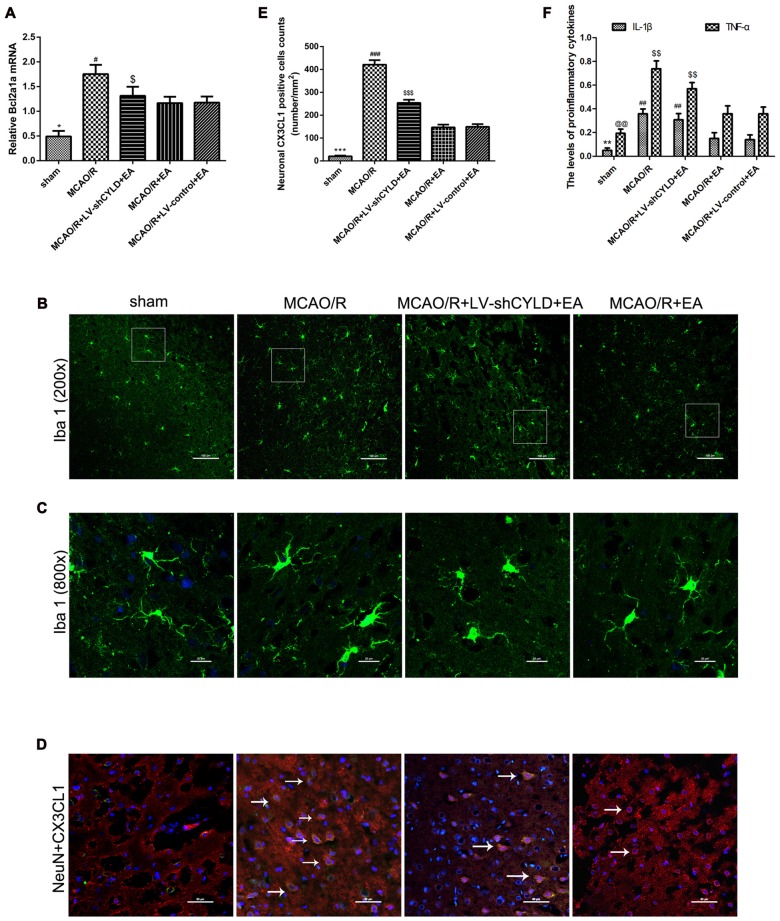
CYLD silencing partially weakens effects of EA on anti-inflammatory injury after 24 h reperfusion.** (A)** Bcl2a1a (microglia) mRNA was measured with RT-qPCR in the peri-ischemic cortices of MCAO/R, MCAO/R + LV-shCYLD + EA and MCAO/R + EA group rats and the sham cortices. β-actin was a loading control. *p < 0.001 vs. MCAO/R group, ^#^*p* < 0.05, ^$^*p* < 0.001 vs. MCAO/R + EA group, *n* = 6/group. **(B)** Immunofluorescence staining showed that CYLD silencing partially weakened the effect of EA on repressing activated microglia (Iba1, green), *n* = 9/group (Scale bar = 100 μm). **(C)** Magnification of **(B)** shows activated morphology of microglia (Scale bar = 20 μm). **(D)** Neuronal CX3CL1 positive cells (white arrows) of the peri-ischemic cortex from merged image (co-expression of neurons and CX3CL1) after ischemic stroke (Scale bar = 50 μm). **(E)** Neuronal CX3CL1 positive cell counts were expressed as number/mm^2^. ***p < 0.001 vs. MCAO/R group, ^###^*p* < 0.05, ^$$$^*p* < 0.001 vs. MCAO/R + EA group.** (F)** IL-1β and TNF-α were detected using ELISA. **p < 0.05, ^@@^*p* < 0.001 vs. MCAO/R group, ^##^*p* < 0.05, ^$$^*p* < 0.05 vs. MCAO/R + EA group, *n* = 5/group.

Microglia are the pivotal immune defense in the CNS. The activation of microglia plays an essential role during the acute stage of focal cerebral ischemia/reperfusion. Therefore, the level of Bcl2a1a mRNA which is the marker of microglia activation was detected by RT-qPCR and the morphology of microglia was investigated by using double-immunofluorescence labeling staining after 24 h reperfusion. Rats were randomly divided into five groups: sham, MCAO/R, MCAO/R + EA, MCAO/R + LV-shCYLD + EA, MCAO/R + LV-control + EA. RT-qPCR showed that Bcl2a1a mRNA in the MCAO/R group was higher than that in sham group (Figure 6A) but Bcl2a1a mRNA in the MCAO/R + EA group was decreased compared with the MCAO/R group (Figure 6A). Bcl2a1a mRNA was significantly higher in the MCAO/R + LV-shCYLD + EA group compared with MCAO/R + EA group (Figure 6A). There was no significant difference between the MCAO/R + LV-control + EA and MCAO/R + EA groups (Figure 6A). Double-immunofluorescent staining showed that microglia present the resting state with a ramified morphology in shams (Figures 6B,C). There were more activated microglia with larger cell bodies and retracted, thickened protuberances in the ischemic border region in the MCAO/R group than in shams (Figure 6C). In addition, there were fewer activated microglia in the MCAO/R + EA group than in the MCAO/R group (Figures 6B,C). However, there were more activated microglia cells in the MCAO/R + LV-shCYLD + EA group compared with the MCAO/R + EA group (Figures 6B,C). These demonstrate that CYLD silencing partially weakened the effect of EA on repressing activated microglia. In addition, Neuronal CX3CL1 leads to activation of microglia. To explore the effect of EA on repressing CX3CL1 expression via upregulating neuronal CYLD. Thus, CX3CL1 expression was assessed by using double-immunofluorescent staining. There was less neuronal CX3CL1 positive cells in the MCAO/R + EA group than in the MCAO/R group (Figures 6D,E). Compared with MCAO/R + EA treatment, more neuronal CX3CL1 positive cells were found in the peri-ischemic cortices of the MCAO/R + LV-shCYLD + EA group (Figures 6D,E).

The levels of TNF-α and IL-1β in the border region of the ischemic cortex were tested after 24 h reperfusion using ELISA. TNF-α and IL-1β in the ischemic cortical border region in the MCAO/R group were higher than in sham group (Figure 6F). Similarly, TNF-α and IL-1β decreased after EA in the MCAO/R + EA group compared with the MCAO/R group (Figure 6F). Pro-inflammatory cytokines were increased in the MCAO/R + LV-shCYLD + EA group compared with the MCAO/R + EA group (Figure 6F). There was no significant difference observed between MCAO/R + EA and MCAO/R + LV-control + EA groups (Figure 6F). CYLD silencing partially weakens anti-inflammatory effects of EA so EA may repress excessive activation of microglia and reduce deleterious pro-inflammatory cytokines in part by upregulating CYLD after focal cerebral ischemia/reperfusion.

### Upregulation of Neuronal CYLD Is Essential for EA-induced Inhibition of NF-κB Activation after Focal Cerebral Ischemia/Reperfusion

**Figure 7:**
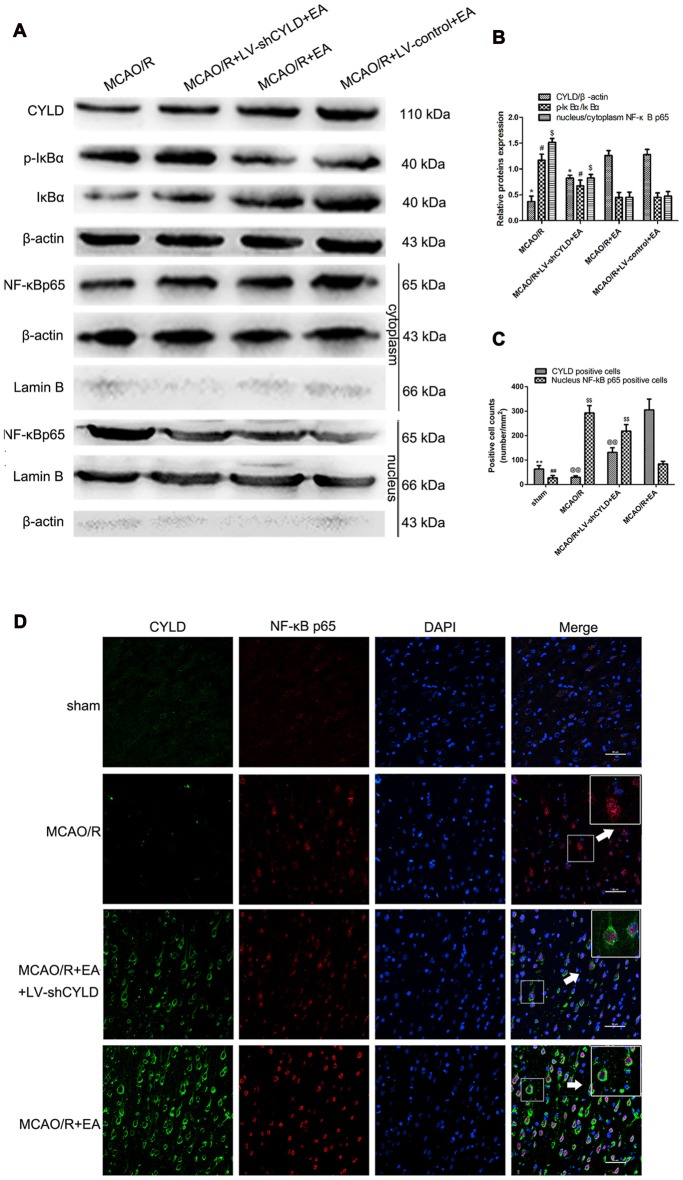
EA inhibited NF-κB signaling pathway by upregulating CYLD.** (A)** Western blot of CYLD, p-IκBα, IκBα, nuclear and cytoplasmic NF-κB p65 expression after 24 h reperfusion. Total and cytoplasm proteins normalized with β-actin and nuclear protein normalized with lamin B. **(B)** CYLD expression, ratio of p-IκBα and nuclear translocation ratios of NF-κB p65. *p < 0.001, ^#^*p* < 0.05, ^$^*p* < 0.001 vs. MCAO/R + EA group, *n* = 5/group.** (C)** Neuronal CYLD and nuclear NF-κB p65 positive cells counts are presented as number/mm^2^. **p < 0.001, ^##^*p* < 0.05 vs. MCAO/R group, ^@@^*p* < 0.05, ^$$^*p* < 0.05 vs. MCAO/R+EA group, *n* = 5/group. **(D)** Immunofluorescent co-expression of CYLD (green) and NF-κB p65 (red) after 24 h reperfusion in borders of ischemic areas (Scale bar = 50 μm). White arrows indicated magnified components from merged image, *n* = 5/group.

As mentioned above, activation of NF-κB signaling pathway is the key step in the inflammatory response after focal cerebral ischemia/reperfusion. We confirmed whether the neuronal CYLD was essential for the effect of EA on anti-inflammatory injury via inhibiting the NF-κB signaling pathway. Rats were randomly divided into four groups included MCAO/R, MCAO/R + LV-shCYLD + EA, MCAO/R + EA and MCAO/R + LV-control + EA. After 24 h reperfusion, Western blot showed increased CYLD protein and less p-IκBα/IκBα in the MCAO/R + EA group compared with the MCAO/R group (Figures 7A,B). CYLD protein decreased and p-IκBα/IκB increased with MCAO/R + LV-shCYLD + EA compared to MCAO/R + EA (Figures 7A,B). There was less nuclear translocation ratio of NF-κB p65 in the MCAO/R + EA group compared with the MCAO/R group (Figures 7A,B). More nuclear translocation of NF-κB p65 was found in the MCAO/R + LV-shCYLD + EA group compared with MCAO/R + EA group (Figures 7A,B).

We found that NF-κB p65 was mainly regulated in neurons by EA in our previous study (Zhan et al., 2016). In addition, CYLD expression was mainly located in neurons. To explore neuronal CYLD expression is required for the effect of EA on inhibiting NF-κB p65 nuclear translocation. Therefore, double-immunofluorescent staining was used to observe co-expression between CYLD and NF-κB p65 in sham, MCAO/R, MCAO/R + LV-shCYLD + EA and MCAO/R + EA groups. After 24 h reperfusion, co-expression between CYLD and NF-κB p65 was measured and CYLD immunopositive cells and cytoplasmic NF-κB p65 were increased with MCAO/R + EA compared with the MCAO/R group (Figures 7C,D). There were fewer CYLD immunopositive cells and more NF-κB p65 nuclear translocation with MCAO/R + LV-shCYLD + EA than in the MCAO/R + EA group. CYLD silencing partially weakened the effect of EA (Figures 7C,D). Thus, neuronal CYLD was essential for EA-induced inhibition of the NF-κB signaling pathway after ischemic stroke.

## Discussion

Inflammation is a consequence of focal cerebral ischemia/reperfusion and contributes to neuronal death (Iadecola and Alexander, 2001; Vidale et al., 2017). The NF-κB signaling pathway is a key regulator of inflammatory responses and CYLD may negatively regulate IKK complex activation to inhibit the NF-κB signaling pathway via deubiquitinating IKKγ (Glittenberg and Ligoxygakis, 2007; Courtois, 2008; Legarda-Addison et al., 2009; Shifera, 2010b; Gerlach et al., 2011; Massoumi, 2011; Tokunaga, 2013). However, how CYLD modulates acute inflammatory injury induced by ischemic stroke is limited so we studied a rat model of MCAO and measured CYLD expression after focal cerebral ischemia/reperfusion.

CYLD was found constitutively expressed in adult human livers, lungs, spleens, and kidneys (Günthner et al., 2013). However, CYLD reports in the brain after ischemic stroke are few. We found CYLD mRNA and protein expression in sham rats, which indicated that CYLD may be constitutively expressed in the brain. In addition, at 6 h after reperfusion, CYLD mRNA and protein expression decreased and this may be associated with neuronal damage-induced by focal cerebral ischemia/reperfusion. The least expression was at 24 h after reperfusion, which was followed peak expression after 72 h reperfusion. After kidney ischemia/reperfusion injury, CYLD mRNA is reported to fall at 4 h reperfusion and dropped rapidly at 24 h reperfusion, consistent with our study (Günthner et al., 2013). A few minutes after ischemic stroke, an imbalance exists between abnormal influx of Na^+^ and efflux of K^+^ in neurons, reducing ATP production. Subsequently, decreased ATP reduces reuptake of glutamate which overstimulates glutamate receptors contributing to Ca^+^ influx. When Ca^+^ influx increases ROS increase in neurons rather than glial cells. ROS negatively regulate transcription of CYLD so we suggest that reduced CYLD expression before 48 h may be related and anti-inflammation only depends on endogenous CYLD during the acute phase of focal cerebral ischemia/reperfusion.

CYLD is primarily in the cytoplasm and peri-nuclear spaces of many cell types (Mathis et al., 2015), is expressed in neuronal nuclei and weakly expressed in cytoplasms of adult mice (Magavi et al., 2000). Feng’s group found that CYLD protein was strongly expressed in primary cortical neurons after OGD (Feng et al., 2017). However, they did not identify the cell type expressing CYLD *in vivo* after hypoxia-ischemia. We found that CYLD was mainly expressed in the cytoplasm of cortical neurons of peri-ischemic areas, not in microglia or astrocyte, during the acute stage of focal cerebral ischemia/reperfusion. Likely CYLD is connected with IKKγ in the cytoplasm of neurons. We speculated that the location of CYLD is associated with cell status and NF-κB expression was observed in cortical neurons after cerebral ischemia, suggesting an anti-inflammatory role for CYLD. Thus, neuronal CYLD may contribute to the anti-inflammatory process via immune cells via a specific signaling pathway in the peri-ischemic cortical area. We report that CYLD overexpression was neuroprotective, reduced infarct volume and decreased pro-inflammatory cytokines and that CYLD silencing exacerbated neurological deficits and increased infarct volumes and inflammatory injury. Thus, neuronal CYLD may be a promising potential therapeutic target for the acute stage ischemic brain injury.

EA is reported to reduce cerebral hypoperfusion-induced cognitive deficits and hippocampal LTP inhibition via promoting dopamine and its major metabolite release and D1/D5Rs activation (Ye et al., 2017). In addition, EA treatment prevented nuclear translocation of NF-κB p65 and suppressed expression of p38 MAPK and myeloid differentiation factor 88 (MyD88) in the peri-infract sensorimotor cortex during ischemic stroke (Liu et al., 2016a). EA may reduce inflammatory injury via NF-κB signaling during ischemic stroke (Kavoussi and Ross, 2007) as we noted that EA significantly inhibited activation of NF-κB, activated microglia, and pro-inflammatory cytokines to reduce inflammatory injury after ischemic stroke but how this occurs is uncertain.

EA upregulated CYLD mRNA and protein expression in the peri-ischemic cortex after ischemia/reperfusion but EA caused a substantial drop in CYLD protein at 6 h reperfusion. CYLD is also associated the p38 MAPK signaling pathway. The p38/CYLD pathway is involved in neuronal necroptosis induced by OGD injury and the p38/CYLD pathway can be activated to increase CYLD protein expression. Also, EA inhibited the p38 MAPK signaling pathway in the hippocampus of rats with cerebral ischemia/reperfusion injury and EA reduces CYLD protein at 6 h reperfusion, which may be associated with p38 MAPK signaling. In addition, EA increased the number of neuronal CYLD-positive cells in the ischemic cortical border region at 24 h reperfusion. Thus, EA upregulated expression of CYLD in peri-ischemic areas after focal cerebral ischemia/reperfusion. Moreover, CYLD silencing partially weakened the impact of EA for improving neurological function and repressing infarct volume, activated microglia, and the production of TNF-α and IL-1β and on regulating the interaction between neurons and microglia by suppressing neuronal CX3CL1. Therefore, EA may reduce inflammatory injury by upregulating CYLD.

Mutation or disruption of the activity of CYLD in animals aggravates acute and chronic liver injury (Hellerbrand and Massoumi, 2016). In addition, lasting activation of NF-κB due to CYLD mutations has been observed in multiple myeloma (Demchenko et al., 2010). Moreover, a recent study demonstrated that blocking Notch signaling in myeloid cells reduced hepatic ischemia reperfusion injury by repressing activation of NF-κB through enhancing CYLD (Yu et al., 2016). We report that EA inhibited degradation of IκBα and NF-κB nuclear translocation by increasing CYLD. CYLD and NF-κB p65 were co-expressed in cortical neurons of the peri-ischemic area and more CYLD positive cells were observed there, but NF-κB p65 was primarily maintained in the cytoplasm after EA treatment during the acute stage of ischemia/reperfusion in non-silenced CYLD rats. This may indicate that upregulation of neuronal CYLD by EA has an anti-inflammatory role in ischemic stroke. Although it is biphasic that inflammation exerts beneficial or detrimental impacts on brain tissues after the ischemic stroke. M1 type microglia, which are detrimental to brain tissues, predominate in the peri-ischemic areas during the first week (Nayak et al., 2014). Therefore, it is important that reducing inflammatory injury in the border of ischemic areas during the acute stage. However, it is possible that overexpression of CYLD leads to immune inhibition if excessively regulated. Thus, moderate regulation must occur based on temporal characteristics. In addition, CYLD inhibited the NF-κB signaling pathway via deubiquitinating IKKγ (Bignell et al., 2000) and CYLD activity is tightly controlled by phosphorylation, which is a negative feedback loop (Sun, 2010). Phosphorylation of CYLD was blocked in IKKγ-deficient cells and rescued with IKKγ reconstitution (Reiley et al., 2005). CYLD expression was increased by EA so we speculate that the negative feedback may control CYLD activity over time and this may inhibit excessive regulation.

Thus, CYLD, chiefly expressed in cortical neurons of the peri-ischemic area, is neuroprotective and anti-inflammatory during focal cerebral ischemia/reperfusion in rats. In addition, upregulating neuronal CYLD expression by EA may prevent NF-κB nuclear translocation and decrease neuronal CX3CL1 expression, inducing subsequent inhibition of microglial activation and pro-inflammatory cytokines in peri-ischemic areas.

## Author Contributions

JJ designed the study, performed all the experiments, acquired and analyzed the data, performed the statistical analysis and drafted the manuscript. YL handled the funding, gave essential advice on the design, supervised all the experiments and provided the critical revision of the manuscript. WQ handled the funding and participated in the design of the study. HM, QL, JZ and YZ contributed reagents and materials.

## Conflict of Interest Statement

The authors declare that the research was conducted in the absence of any commercial or financial relationships that could be construed as a potential conflict of interest.
