# Supplementary material for: Electroacupuncture Suppresses the NF-κB Signaling Pathway by Upregulating Cylindromatosis to Alleviate Inflammatory Injury in Cerebral Ischemia/Reperfusion Rats
Source: Front Mol Neurosci. 2017 Nov 6;10:363. doi: 10.3389/fnmol.2017.00363 (PMC5681846; doi:10.3389/fnmol.2017.00363)
Supplement: Supplementary file 1 [file Data_Sheet_1.doc]

***Supplementary Material:***

Jin Jiang, Yong Luo^*^

^*^Correspendence: Yong Luo

Author Name: Jin Jiang, Yong Luo*, Wenyi Qin, Hongmei Ma, Qiongli Li, Jian Zhan, Ying Zhang

luoyong1998@163.com

**1. SUPPLEMENTARY TABLE AND FIGURE**

**1.1 Table:**

| Test | Score | | | |
| --- | --- | --- | --- | --- |
|  | 0 | 1 | 2 | 3 |
| Spontaneous activity (in cage for 5 min) | No movement | Barely moves | Moves but does not approach at least three sides of cage | Moves and approaches at least three sides of cage |
| Symmetry of movements (four limbs) | Left side: no movement | Left side: slight movement | Left side: moves slowly | Both sides: move symmetrically |
| Symmetry of forelimbs (outstretching while held by tail) | Left side: no movement, no outreaching | Left side: slight movement to outreach | Left side: moves and outreaches less than right side | Symmetrical outreach |
| Climbing wall of wire cage | --- | Fails to climb | Left side is weak | Normal climbing |
| Reaction to touch on either side of trunk | --- | No response on left side | Weak response on left side | Symmetrical response |
| Response to vibrissae touch | --- | No response on left side | Weak response on left side | Symmetrical response |

**Table S1.** The 18 points system for neurological scores

**1.2 Figures:**

**
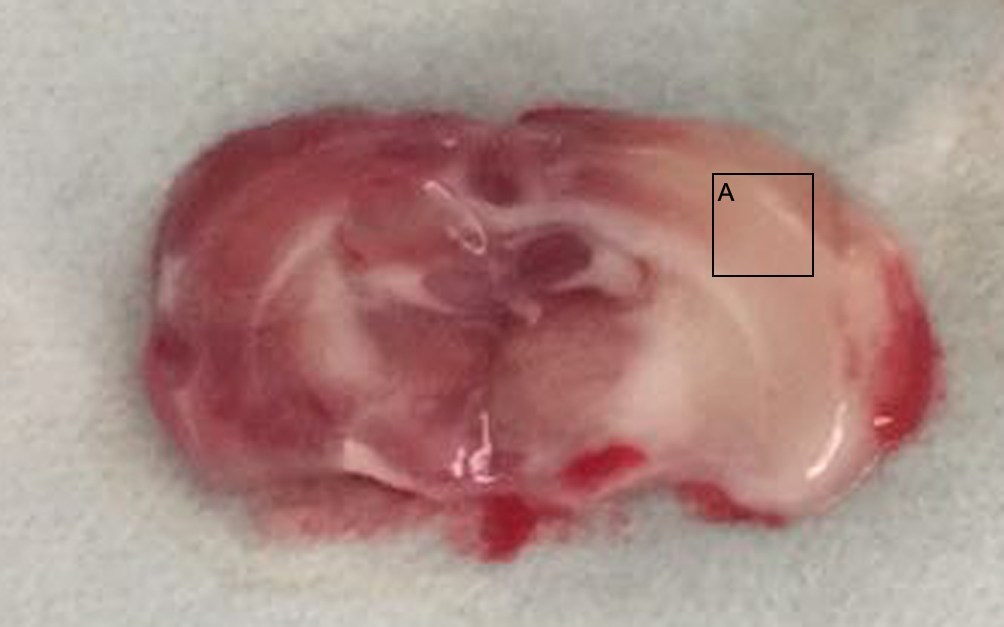
**

**Figure S1.** TTC staining indirectly showing the peri-ischemic area from rats 24 h after MCAO/R. Total RNA and proteins were extracted from the peri-ischemic area (A). The border area (A) was also measured with double-immunofluorescent labeling.

**
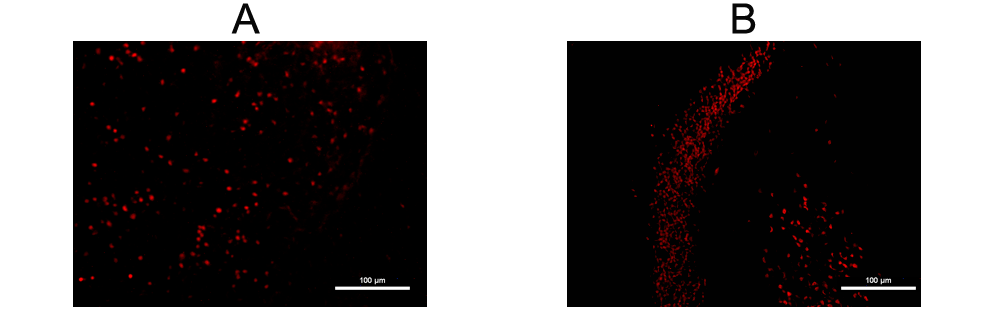
**

**Figure S2.** Spatial expression of CYLD in brain tissues. CYLD expression was found in cortex (A) and hippocampus (B) of sham brains (Scale bar=100 μm).
