# Supplementary material for: Electroacupuncture Suppresses the NF-κB Signaling Pathway by Upregulating Cylindromatosis to Alleviate Inflammatory Injury in Cerebral Ischemia/Reperfusion Rats
Source: Front Mol Neurosci. 2017 Nov 6;10:363. doi: 10.3389/fnmol.2017.00363 (PMC5681846; doi:10.3389/fnmol.2017.00363)
Supplement: Supplementary file 2 [file Table_2.doc]

***Supplementary Material:***

Jin Jiang, Yong Luo^*^

^*^Correspendence: Yong Luo

Author Name: Jin Jiang, Yong Luo*, Wenyi Qin, Hongmei Ma, Qiongli Li, Jian Zhan, Ying Zhang

luoyong1998@163.com

**1. SUPPLEMENTARY TABLE AND FIGURE**

**1.2 Table：**

| Gene | Accession | Forward | Reverse |
| --- | --- | --- | --- |
| cyld | NM_001017380 | 5’GTTCTATGGGGTTATCCGTTGG3’ | 5’GTTCTATGGGGTTATCCGTTGG3’ |
| Bcl2a1a | NM_133416.1 | 5’AAGCTTCCACAAGAGCAGATTG3’ | 5’CAGCCAGCCAGATTTAGGTTC3’ |
| β-actin | NM_031144.3 | 5’ACGGTCAGGTCATCACTATCG3’ | 5’GGCATAGAGGTCTTTACGGATG3’ |

**Table S2 List of primers used for RT-qPCR**
